# Lactoferrin ameliorates dopaminergic neurodegeneration and motor deficits in MPTP-treated mice

**DOI:** 10.1016/j.redox.2018.101090

**Published:** 2018-12-21

**Authors:** Shuang-Feng Xu, Yan-Hui Zhang, Shan Wang, Zhong-Qiu Pang, Yong-Gang Fan, Jia-Yi Li, Zhan-You Wang, Chuang Guo

**Affiliations:** a College of Life and Health Sciences, Northeastern University, No.195, Chuangxin Road, Hunnan District, Shenyang 110169, China; b Institute of Health Sciences, Key Laboratory of Medical Cell Biology of Ministry of Education, China Medical University, Shenyang 110122, China; c Neural Plasticity and Repair Unit, Wallenberg Neuroscience Center, Department of Experimental Medical Science, Lund University, BMC A10, 22184 Lund, Sweden

**Keywords:** Parkinson's disease, Iron chelators, Lactoferrin, Motor dysfunction

## Abstract

Brain iron accumulation is common in patients with Parkinson's disease (PD). Iron chelators have been investigated for their ability to prevent neurodegenerative diseases with features of iron overload. Given the non-trivial side effects of classical iron chelators, lactoferrin (Lf), a multifunctional iron-binding globular glycoprotein, was screened to identify novel neuroprotective pathways against dopaminergic neuronal impairment. We found that Lf substantially ameliorated PD-like motor dysfunction in the subacute 1-methyl-4-phenyl-1,2,3,6-tetrahydropyridine (MPTP)-induced mouse model of PD. We further showed that Lf could alleviate MPTP-triggered apoptosis of DA neurons, neuroinflammation, and histological alterations. As expected, we also found that Lf suppressed MPTP-induced excessive iron accumulation and the upregulation of divalent metal transporter (DMT1) and transferrin receptor (TFR), which is the main intracellular iron regulation protein, and subsequently improved the activity of several antioxidant enzymes. We probed further and determined that the neuroprotection provided by Lf was involved in the upregulated levels of brain-derived neurotrophic factor (BDNF), hypoxia-inducible factor 1α (HIF-1α) and its downstream protein, accompanied by the activation of extracellular regulated protein kinases (ERK) and cAMP response element binding protein (CREB), as well as decreased phosphorylation of c-Jun N-terminal kinase (JNK) and mitogen activated protein kinase (MAPK)/P38 kinase in vitro and in vivo. Our findings suggest that Lf may be an alternative safe drug in ameliorating MPTP-induced brain abnormalities and movement disorder.

## 1 Introduction

Dysregulation of iron metabolism has been linked to the pathogenesis of several neurodegenerative disorders, including Parkinson's disease (PD). Iron has been shown to be accumulated in substantia nigra pars compacta (SNpc) in PD patients [1], as well as in the brain of the PD mouse model [2]. The positive effect of iron chelator treatment on PD has been investigated by genetic or pharmacological methods and its ability to reduce the iron level and prevent toxicity in the 1-methyl-4-phenyl-1,2,3,6-tetrahydropyridine (MPTP) mouse model of PD had been previously validated [3], [4], [5]. Several iron chelators, such as clioquinol and deferiprone, are considered to be promising drugs for PD treatment; in particular, deferiprone showed the ability to sustain a decreased iron level in the SN and improve the patient condition in several clinical trials [6], [7], [8].

The clinical application of iron chelators has a bright future in PD therapy; however, challenges remain in the identification of an iron chelator that simultaneously exhibits the following four qualities: natural security, low molecular weight, varying affinities for iron and good brain-targeting efficiency [9], [10]. It is therefore necessary to screen an iron chelator with all properties; thus, we turned our attention to lactoferrin (Lf). Lf occurs naturally in human and bovine milk without safety concerns, and its 80-kDa molecular weight contributes to penetrate the blood-brain barrier (BBB). The high affinity for Fe^3+^ and the brain targeting of Lf have also been confirmed [11]. These features make Lf a promising candidate for PD clinical trials. In addition, several other physiological functions, such as immune regulation, antioxidant, anti-inflammation, and anti-apoptosis, can facilitate PD therapy [12].

We recently showed that Lf can retard cognitive impairment in Alzheimer's mice [13], in which the protective mechanism is similar to that of the classic iron chelator deferoxamine (DFO) [14]. It has also been proven that DFO could provide neuroprotective effects against dopaminergic (DA) neuronal impairment via several mechanisms in MPTP-induced PD model mice [4]. Thus, we hypothesized that the supplementation of Lf could correct elevated iron and protect damaged dopaminergic neurons in PD mice. In the present study, we determine the capability of Lf to rescue DA neuron degeneration in MPTP-treated mice and 1-methyl-4-phenylpyridiniumion (MPP^+^)-treated cells. We also address the molecular mechanisms by which Lf ameliorates PD-like pathological features, such as α-Synuclein (α-Syn) accumulation, apoptosis of DA neurons, excessive iron accumulation and neuroinflammation. Specifically, Lf enhanced the expression of brain-derived neurotrophic factor (BDNF) via an extracellular regulated protein kinase (ERK)-cAMP response element binding protein (CREB) pathway and hypoxia-inducible factor 1α (HIF-1α)-dependent mechanism to protect mice against motor dysfunction.

## 2 Materials and methods

### 2.1 Animals and treatments

All male C57BL/6 mice used in this study were provided by the Jackson laboratory (BarHarbor, ME, USA). Thirty 6-month-old C57BL/6 mice were randomly assigned to the Control group (saline-treated group), MPTP-treated group, and MPTP+Lf-treated group. With the exception of the saline-treated group, 30 mg/kg MPTP (Sigma-Aldrich, M0896) was injected into the abdomens of the mice once a day for 5 days to produce an experimental PD model. In the MPTP+Lf-treated group, the MPTP-induced PD mice received human Lf (hLf; Sigma-Aldrich, L4040, 4 mg/kg body weight, dissolved in saline) via peritoneal injection once per day for one week. All animal experimental procedures were approved by the Laboratory of Animal Ethical Committee of China Medical University.

### 2.2 Open field test

The detailed method of the open field test was the same as described previously [15]. According to the experimental requirements, analysis and export of different experimental parameters, such as the 5 min animal movement distance, climbing lattice number, standing times, movement speed, and the central movement distance, were performed.

### 2.3 Pole-climbing test

The pole was divided into two sections, and each section was 25 cm. The climbing times of mice were recorded by the upper and lower two periods and were assigned different scores according to the time: more than 6 s is one point, less than 6 s and more than 3 s is two points, and less than 3 s is three points. Analysis was based on the score.

### 2.4 Traction test

Experimental approach: mice were placed on a horizontal grid. The grid was straight or flipped and the time was recorded that hanging on the grid; in accordance with the standard for evaluation: less than 10 s is zero points, less than 20 s and more than 10 s is one points, less than 30 s and more than 20 s is two points, less than 40 s and more than 30 s is three points, less than 50 s and more than 40 s is four points, less than 60 s and more than 50 s is five points, more than 60 s is six point, different scores were provided and analysis was conducted.

### 2.5 Gait analysis experiment

The white paper, watercolor paint, and brush were prepared in advance. During the experiment, the mice were successfully walked through the white paper passages, leaving the footprints of the mice; the footprints were dried and analyzed. The average variation of the gait distance, the gait distance and the distance between two adjacent left or right footprints, and the gait variability rate are used to reflect the ataxia of the experimental mice.

### 2.6 Iron staining

The iron content was detected by an improved iron staining based on Prussian blue staining, the detailed method was the same as described previously [16], [17].

### 2.7 Culture and treatment of human SH-SY5Y neuroblastoma cells and mouse mesencephalic dopaminergic cell line MN9D cells

Human SH-SY5Y neuroblastoma cells and mouse mesencephalic dopaminergic cell line MN9D cells were cultured as described previously [4], [13]. The drug treatment concentrations of MPP^+^ (4 mM; Sigma-Aldrich, D048), hLf (0.1 mg/ml; Sigma-Aldrich, L4894), SP600125 (5 μM), PD98059 (20 μM), SB203580 (7.5 μM), H89 (1 μM) and YC-1 (20 nM) were based on previously described studies [4], [13]. The culture medium was changed to DMEM without FBS for 12 h prior to the drug treatments. hLf and inhibitors were added to the medium 2 h before MPP^+^ treatment. The cells were collected for Western blot analyses after 24 h.

### 2.8 Immunohistochemistry and immunofluorescence

The immunohistochemistry and immunofluorescence processes were described specifically in our previous experiment [4], [13]. In the present study, for immunohistochemistry, the slice were incubated with rabbit anti-tyrosine hydroxylase (TH, 1:800; Millipore) and rat anti-dopamine transporter (DAT, 1:800; Millipore). For immunofluorescence staining, the mice brain slice were co-incubated with primary antibody TH (1:600; Millipore) and glial fibrillary acidic protein (GFAP, 1:200; Santa Cruz). SH-SY5Y cells and MN9D cells were incubated with microtubule-associated protein-2 (MAP-2, 1:200; Santa Cruz), HIF-1α (1:200; Cell Signaling Tech), and TH (1:400; Millipore). Samples from the mouse brains or cells were observated under a confocal microscope (Leica, DM4000B or Leica, SP8).

### 2.9 Western blot analyses

Extraction all the mice striatum and SN tissues or cells protein, and the procedure and method of Western blots was the same as described previously [4], [16], [18]. The primary antibodies required for this experiment were shown as Table 1. Blots were detected using chemiluminescence imaging analysis system (Tanon, 5500) and enhanced chemiluminescence (ECL) Kits (Tanon, 180–5001).

**Table 1:** List of biological reagents antibody.

**Antibody**	**Source**	**Manufacturer**	**Dilution ratio**
Bax	Rabbit	Santa Cruz	1:1000
Bcl2	Mouse	Santa Cruz	1:1000
BDNF	Rabbit	Cell Signaling Tech	1:1000
caspase3	Rabbit	Cell Signaling Tech	1:1000
p-CREB	Rabbit	Cell Signaling Tech	1:1000
CREB	Rabbit	Cell Signaling Tech	1:1000
DAT	Rat	Millipore	1:3000
DMT1	Rabbit	Abcam	1:2000
p-ERK1/2	Rabbit	Cell Signaling Tech	1:2000
ERK1/2	Rabbit	Cell Signaling Tech	1:2000
Fpn	Goat	Santa Cruz	1:1000
GFAP	Rabbit	Santa Cruz	1:1000
GPX4	Rabbit	Cell Signaling Tech	1:1000
GAP43	Mouse	Cell Signaling Tech	1:2000
HIF-1α	Rabbit	Cell Signaling Tech	1:1000
IBA1	Rabbit	Abcam	1:2000
IL-1β	Rabbit	Santa Cruz	1:1000
p-JNK1/2	Mouse	Cell Signaling Tech	1:2000
JNK1/2	Mouse	Cell Signaling Tech	1:2000
p-NFκB	Rabbit	Cell Signaling Tech	1:1000
NFκB	Rabbit	Cell Signaling Tech	1:1000
p-P38	Rabbit	Cell Signaling Tech	1:2000
P38	Rabbit	Cell Signaling Tech	1:2000
SOD1	Rabbit	Cell Signaling Tech	1:1000
TFR	Mouse	Invitrogen	1:1000
TH	Rabbit	Millipore	1:3000
TNFα	Mouse	Santa Cruz	1:1000
VEGF	Rabbit	Cell Signaling Tech	1:1000
α-Synuclein	Mouse	Thermo Fisher	1:1000
β-actin	Mouse	Sigma-Aldrich	1:10,000

### 2.10 Measurement of reactive oxygen species (ROS) activity

To determine whether Lf treatment decreased oxidative stress in PD model mice, ROS production was measured using 2′, 7′-dichlorofluorescein diacetate (DCFH-DA) according to the manufacturer's protocol (E004, Jiancheng Biology, Nanjing, China). The detailed method was the same as described previously [15].

### 2.11 Statistical analysis

All results were based on at least three experiments and are presented as the mean ± standard deviation (SD). Group differences were analyzed using one-way analysis of variance (ANOVA). The analyses were performed using ImageJ software and GraphPad Prism 5.0 software, and differences were assumed to be highly significant if the probability (p) value was < 0.01 and significant if p < 0.05.

## 3 Results

### 3.1 Lf alleviates MPTP-induced behavior disorder

**Fig. 1:**
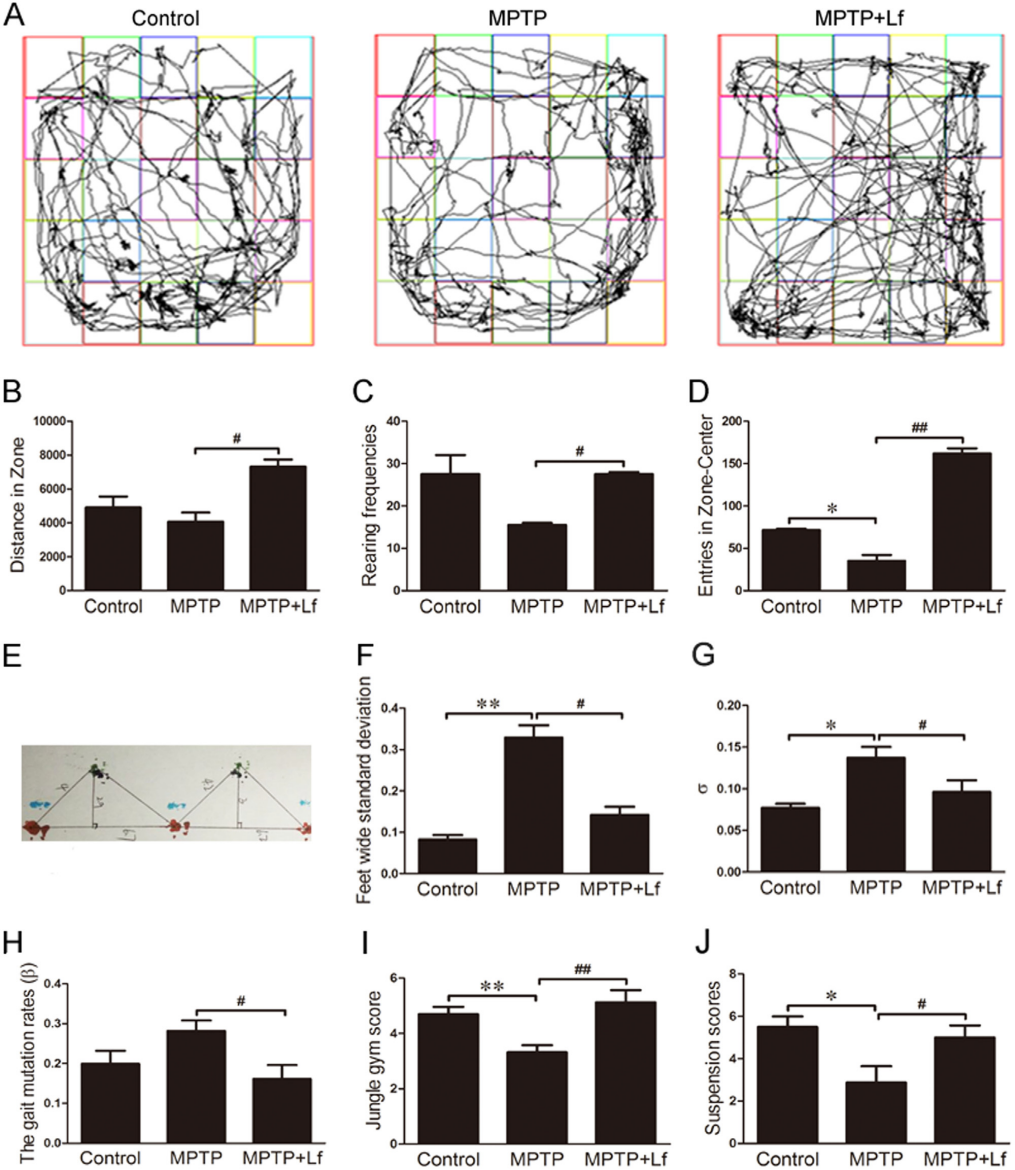
Lf reversed MPTP-induced Parkinsonian dyskinesia. (A) Five minute movement tracks of open field exploration by the mice. (B) Total distance traveled in open field exploration in 5 min (C) Rearing frequencies of open field exploration in 5 min (D) Entries in zone-center showing 5 min of open field exploration by the mice. (E) Gait analysis diagram of gait analysis experiment. (F) Feet wide standard deviation of gait analysis experiment. (G) Average variance of the distance between two adjacent left footprints (σ) of gait analysis experiment. (H) The gait mutation rates (β) of gait analysis experiment. (I) Jungle gym score of pole-climbing test. (J) Suspension scores of traction test. Values are represented as the means ± SD (n = 10). *p < 0.05, **p < 0.01 compared with the Control group; ^#^*p* < 0.05, ^##^*p* < 0.01 compared with the MPTP group.

To determine the effects of Lf administration in the MPTP-induced mouse model of PD, the open field test, climbing pole test, suspension test and gait analysis experiment were assessed. Following a 7-day treatment with hLf, the movement distance and the numbers of both the standing and climbing grid of the MPTP+Lf treatment mice were significantly increased relative to the MPTP group (Fig. 1B-D, p < 0.05 or p < 0.01, respectively), which suggests that the motor activity, curiosity to a fresh environment, anxiety and other emotions of the PD model mice were stronger after hLf administration.

Based on the analysis of Fig. 1E, in the MPTP group mice, the step width, the average distance between adjacent two left footprints or right footprints variation (σ), and the gait mutation rate were significantly increased compared with the control group (Fig. 1F-H, p < 0.01 or p < 0.05, respectively). However, compared with the MPTP group, these indicators were all substantially decreased in the MPTP+Lf group (p < 0.01, Fig. 1F-H), which indicates that the masonic movement ability disorder was alleviated after the administration of hLf.

We further assessed the effects of Lf on dyskinesia with the climbing pole test and suspension test. As shown in Fig. 1I, the reduced climbing score in the MPTP-induced PD mice was significantly increased in the hLf-treated PD mice (p < 0.01, Fig. 1I). Moreover, in the suspension test, the mice in the MPTP group had a shorter suspension time and a significantly lower score than the control mice, whereas the suspension time in the MPTP+Lf group was longer than in the MPTP group; thus, the scores increased after hLf treatment (p < 0.05, Fig. 1J). These results indicated that Lf reversed MPTP-induced Parkinsonian dyskinesia.

### 3.2 Lf decreases MPTP-induced dopaminergic neurons degeneration

**Fig. 2:**
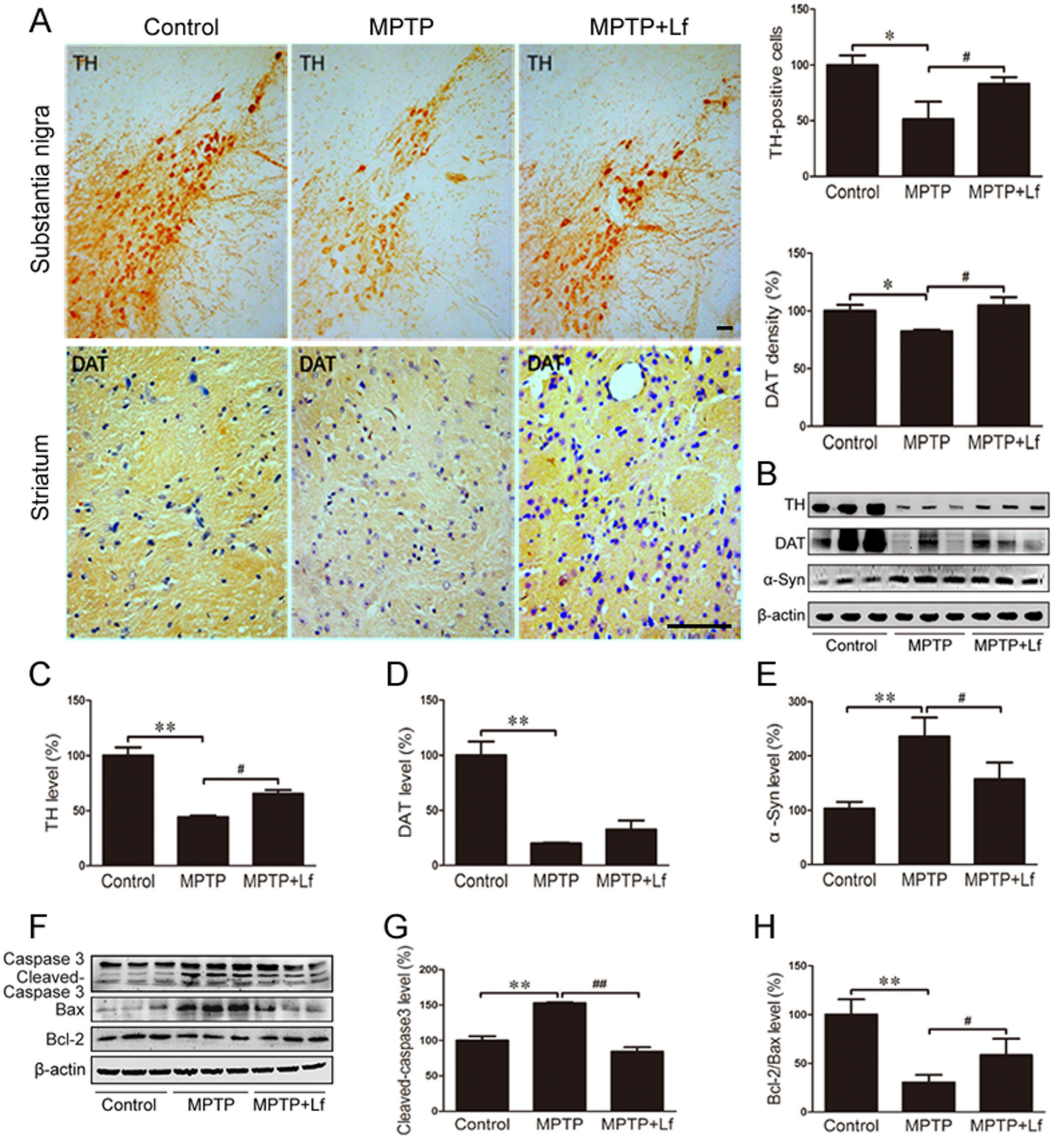
Lf eases apoptosis of dopaminergic neurons induced by MPTP in mice. (A) The levels of TH and DAT were measured by immunostaining analysis. Scale bars = 100 µm, 25 µm. (B-E) The expressions of TH, DAT and α-Syn were determined by Western blot analyses. (F-H) Western blot results showed the protein expression levels of caspase3, Bax and Bcl-2 of three groups of mice and the quantitative analysis chart. Values are represented as the means ± SD (n = 10). *p < 0.05, **p < 0.01 compared with the Control group; ^#^*p* < 0.05, ^##^*p* < 0.01 compared with the MPTP group.

To determine whether Lf treatment protects against nigrostriatal DA neuron lesions induced by MPTP, TH and DAT in the SN and striatum region were explored by immunostaining and Western blot. As shown in Fig. 2A (p < 0.05), hLf administration for seven consecutive days substantially reduced the MPTP-triggered loss of DA neurons and nerve fibers. Western blotting was applied to verify the immunohistochemical findings (p < 0.05, Fig. 2B-D). As shown in Fig. 2B, E, however, MPTP treatment significantly increased the expression of α-Syn, and hLf prevented the increase (p < 0.05, Fig. 2E) in the nigrostriatal area of the MPTP-treated PD mice. These results demonstrated hLf treatment attenuated the MPTP induced DA neuron damage.

MPTP is known to mediate apoptosis and lead to the loss of DA neurons in the SN [19], [20]. Thus, we explored the effect of Lf on the expression of caspase3 among the three treatment groups. Cleaved-caspase3 significantly increased in the brains of the MPTP-treated mice, whereas hLf treatment significantly restored the increase (p < 0.01, Fig. 2F, G). Consistent with caspase3, in the comparison of the MPTP group with the control group, the Bcl-2/Bax ratio was significantly reduced (p < 0.01, Fig. 2F, H), whereas hLf treatment alleviated the decreased ratio (p < 0.05, Fig. 2F, H). These results suggested that a one-week intraperitoneal injection of hLf can effectively inhibit MPTP-induced apoptosis. Together, these results indicated that Lf may be beneficial to DA neuronal survival.

### 3.3 Lf inhibits inflammatory reaction of Glial cells in mice

**Fig. 3:**
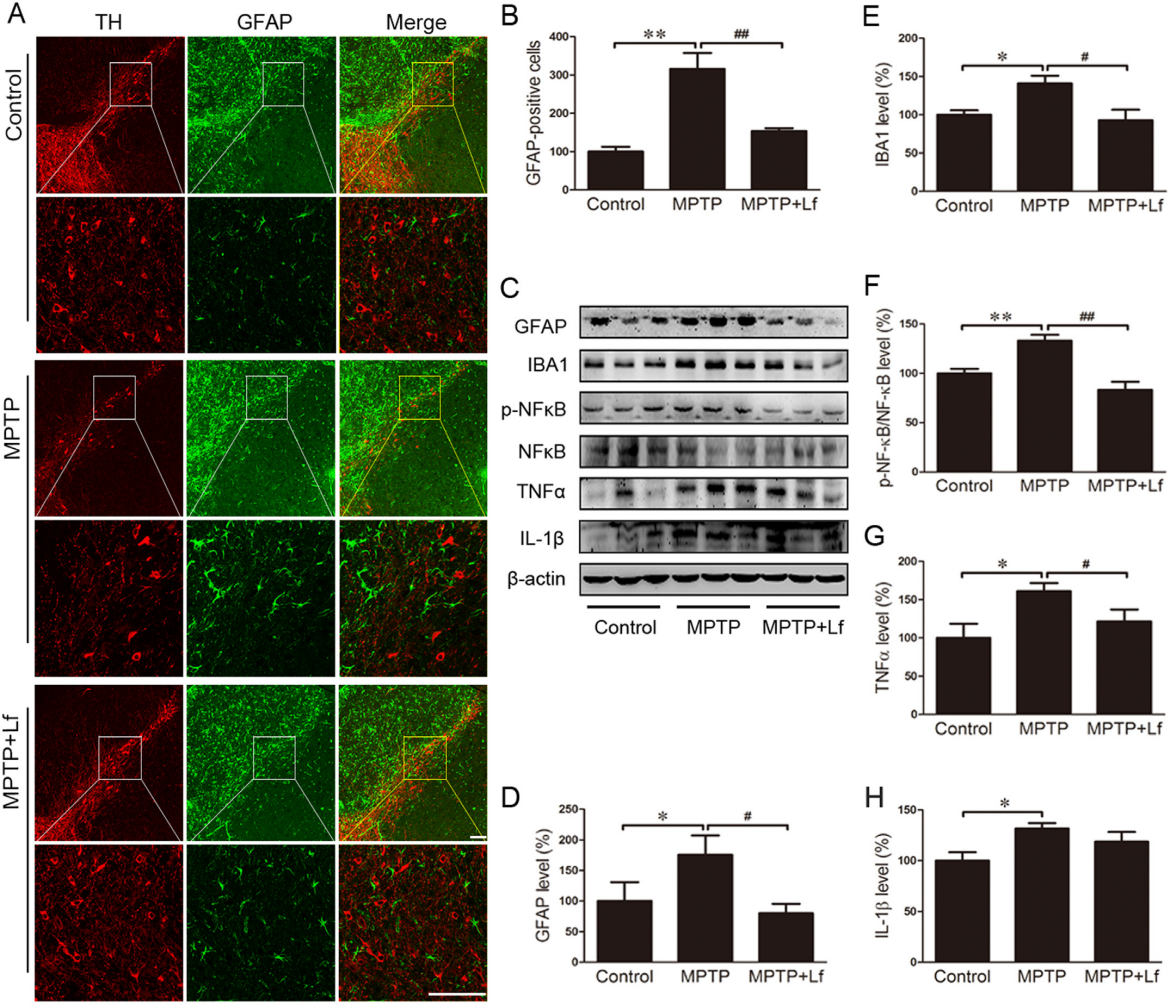
Effects of Lf on glial activation and inflammatory reaction in PD mouse brains. (A, B) Paraffin sections were stained with GFAP in the SN to investigate astrocyte activation and perform a quantitative analysis. Scale bars = 100 µm, 25 µm. (C-H) GFAP, IBA1, p-NFκB, NFκB, TNFα and IL-1β levels were detected by Western blotting and quantitative analysis. Values are represented as the means ± SD (n = 10). *p < 0.05, **p < 0.01 compared with the Control group; ^#^*p* < 0.05, ^##^*p* < 0.01 compared with the MPTP group.

GFAP can be used as a molecular marker for activated astrocytes, and its overexpression has been linked to injury or inflammation [21]. We examined the expression levels of GFAP in the SN via immunofluorescence to further ascertain the activation of astrocytes in the mouse brain (Fig. 3A). We observed that the GFAP production following MPTP treatment significantly increased compared to the control mice, and hLf had a marked inhibition on this increase (p < 0.01, Fig. 3A, B). We also examined the expression levels of GFAP and IBA1 by Western blot, and the results of the expression levels of GFAP were consistent with immunofluorescence (p < 0.05, Fig. 3C, D). In the MPTP mice, the level of IBA1 was significantly increased compared with the controls (p < 0.05, Fig. 3C, E), and the expression level of IBA1 was significantly decreased after hLf treatment in the PD model mice (p < 0.05, Fig. 3C, E).

There are many aspects, such as inflammation, immune responses, cell proliferation, and apoptosis, involved in the function of NFκB. The transcription and expression of pro-inflammatory cytokines, such as TNFα, IL-1β, and IL-6, could be induced by NFκB [22], [23]. MPTP treatment significantly increased the expression of p-NFκB (p < 0.01, Fig. 3C, F), and hLf inhibited this significant increase when compared to the MPTP mice (p < 0.01, Fig. 3C, F). In addition, we investigated the expression levels of TNFα and IL-1β by Western blot; as shown in Fig. 3C, the expression levels of TNFα and IL-1β were significantly increased compared with the control mice, and after hLf treatment, the expression levels of TNFα were significantly decreased (p < 0.05, Fig. 3G); however, the decrease of IL-1β was not significant (P > 0.05, Fig. 3H). As indicated by the results, Lf can inhibit the inflammatory cascade after MPTP intoxication.

### 3.4 Effects of Lf on the distribution of iron, antioxidant ability, and upregulation of HIF-1α and its downstream proteins

**Fig. 4:**
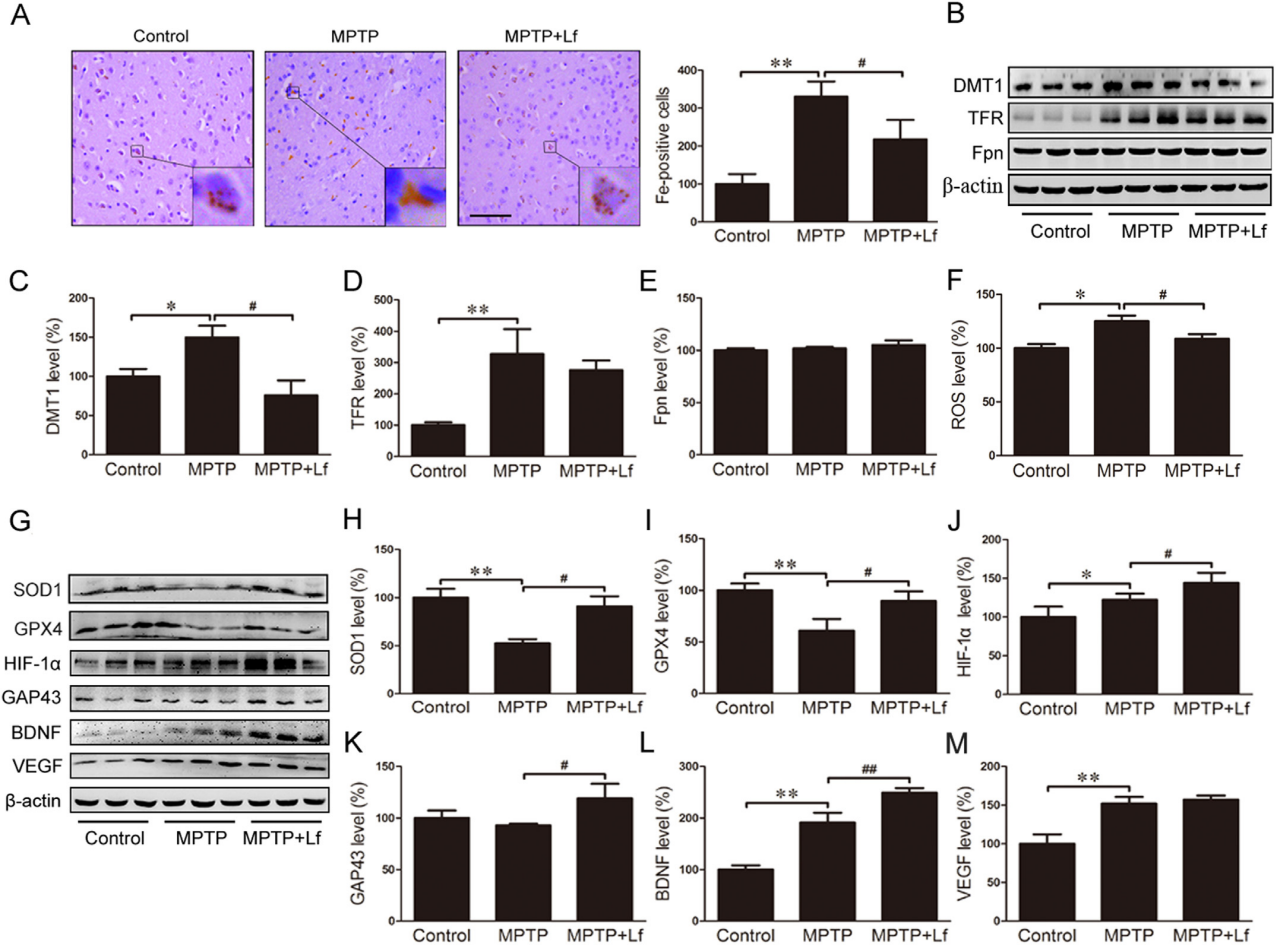
Protective effects of Lf on the distribution of iron and oxidative stress occurred through upregulation of HIF-1α and its downstream proteins. (A) Modified Prussian DAB staining showed a clear change in the iron distribution among three treatments and the quantitative analysis of DAB staining in mouse brains. The brown granules represent iron deposition. Scale bar = 25 µm. (B-E) Western blot results showed the protein expression levels of DMT1, TFR and Fpn of three groups of mice and the quantitative analysis chart. (F) ROS production was detected in the SN of each group by the DCF-DA method. (G) Changes in expression levels of SOD1, GPX4, HIF-1α, GAP43, BDNF and VEGF in three groups of mice by Western blots. Quantitative analysis of the proteins shown in H-M. Values are represented as the means ± SD (n = 10). *p < 0.05, **p < 0.01 compared with the Control group; ^#^*p* < 0.05, ^##^*p* < 0.01 compared with the MPTP group.

The ability of Lf to bind to Fe^3+^ has been reported many times [24], [25]. However, whether Lf treatment could decrease the iron content of DA neurons in the PD mouse model remains unclear. In the present study, we first examined the iron distribution in the SN of the three groups of mice using Perl's DAB staining and analysis. As shown in Fig. 4A, there were more brown granules that iron-positive cells in the neuronal cytoplasm in the SN of the PD model mice than in the controls, and after hLf treatment, the iron-positive cells were substantially lower than in the MPTP mice, which suggests hLf supplementation prevents MPTP-induced iron elevation.

To determine the mechanisms involved in the reduction in the iron content induced by hLf in the PD model mice, we examined three major iron metabolism proteins, DMT1, TFR and Fpn, in the SN and striatum by Western blotting (Fig. 4B). The expressions of DMT1 and TFR in the MPTP group were significantly higher than those in the control group. After hLf treatment, the expression of DMT1 was substantially decreased (p < 0.05, Fig. 4C); however, the decrease of TFR was not significant (p > 0.05, Fig. 4D). There was no significant difference in the Fpn expression in the SN and striatum between the mice treated with MPTP only and the mice treated with hLf and MPTP (p > 0.05, Fig. 4E). These findings suggested that Lf could reduce the iron intake of DA neurons by chelating.

It is generally known that antioxidants play a protective role in defensing free-radical damage in PD [26]. Thus, we investigated the ROS level in the SN, the result showed that MPTP treatment increased the ROS level compared to the controls, and after hLf treatment, the levels of ROS were significantly decreased (p < 0.05, Fig. 4F). Then, we also examined the effects of Lf on several antioxidant enzymes by Western blot. MPTP caused a substantial reduction in the SOD1 and GPX4 expressions, and hLf clearly increased the expressions of SOD1 and GPX4 (p < 0.01, p < 0.05, Fig. 4G-I). These findings showed that Lf could protect the brain by increasing the antioxidant contents.

The relationship between iron chelators and HIF-1α has been validated by our previous laboratory study [4], [13]. There is a potential role of HIF-1α protein in neuroprotection and the regulation of TH and α-Syn [17], [27]. To investigate the effect of Lf on the expression of HIF-1α, immunoblotting analysis was performed to quantify its expression level. The results showed that MPTP increased the HIF-1α expression compared to the control group, and hLf treatment induced a significant upregulation of HIF-1α compared to the MPTP group (p < 0.05, Fig. 4G, J).

GAP43 is mainly embodied in the role of neurotrophic factors that promote nerve growth, development and synaptic connections [28], [29], [30]. As shown in Fig. 4G, K, the expression levels of GAP43 in the MPTP mice were lower than those in the hLf-treated PD mice (p < 0.05, Fig. 4K), which indicates that hLf treatment improved brain recovery. BDNF is a protein synthesized in the brain and plays an important role in the central nervous system development process, survival of neurons, differentiation, growth and development [31]. Our results showed that MPTP increased BDNF expression compared to the control mice (p < 0.01, Fig. 4G, L), and hLf treatment induced a significant upregulation of BDNF compared to the MPTP mice (p < 0.01, Fig. 4G, L). VEGF is a multidimensional growth factor that stimulates homologous receptor-mediated multiple functions in endothelial cells [32]. The results showed that MPTP significantly increased the VEGF expression compared to the control group (p < 0.01, Fig. 4G, M). After hLf treatment, the expression level of VEGF did not achieve a significant change compared to the MPTP mice (P > 0.05, Fig. 4G, M). Thus, we speculated Lf could protect DA neurons by upregulating the expression levels of HIF-1α and its downstream proteins.

### 3.5 Effects of Lf on MAPK signaling pathways in mice

**Fig. 5:**
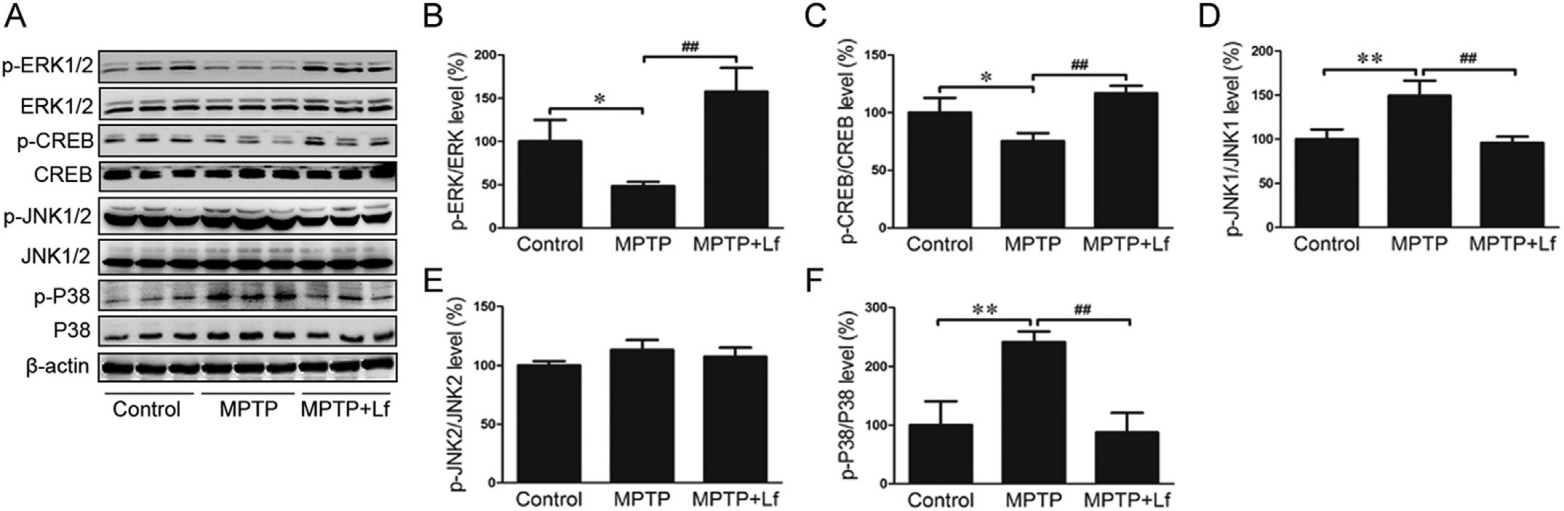
Effects of Lf on MAPK signaling pathways in mice. (A) Changes in expression levels of p-ERK1/2, ERK1/2, p-CREB, CREB, p-JNK1/2, JNK1/2, p-p38 and p38 in three groups of mice by Western blots. Quantitative analysis of the proteins shown in B-F. Values are represented as the means ± SD (n = 10). *p < 0.05, **p < 0.01 compared with the Control group; ^#^*p* < 0.05, ^##^*p* < 0.01 compared with the MPTP group.

MAPK and linked kinase pathways have been implicated in several neurodegenerative diseases, including PD. The ERK MAPK signaling pathway plays an important role in growth factor-mediated cell proliferation [33] and is also involved in upregulating HIF-1α and BDNF expression [34], [35]. To confirm whether Lf acted on the MAPK signaling pathway, we performed Western blot to estimate these protein markers. For the p-ERK1/2 and p-CREB protein, the expression levels were decreased by MPTP treatment (p < 0.05, Fig. 5A-C); however, hLf induced a significant increase compared to the MPTP mice (p < 0.01, Fig. 5A-C). Moreover, the expression levels of p-JNK1/2 and p-P38 significantly increased following MPTP treatment (p < 0.01, Fig. 5A, D-F), whereas treatment with hLf clearly inhibited this increase (p < 0.01, Fig. 5A, D-F). The expression levels of the ERK1/2, CREB, JNK1/2, and P38 proteins did not differ among the groups. These results indicated that Lf could protect DA neurons by activating the ERK MAPK signaling pathways, and at the same time, its mechanism is correlated with the inhibition of JNK and P38.

### 3.6 The key roles of the MAPK signaling pathway in Lf-blocked MPP^+^-induced SH-SY5Y cell death

**Fig. 6:**
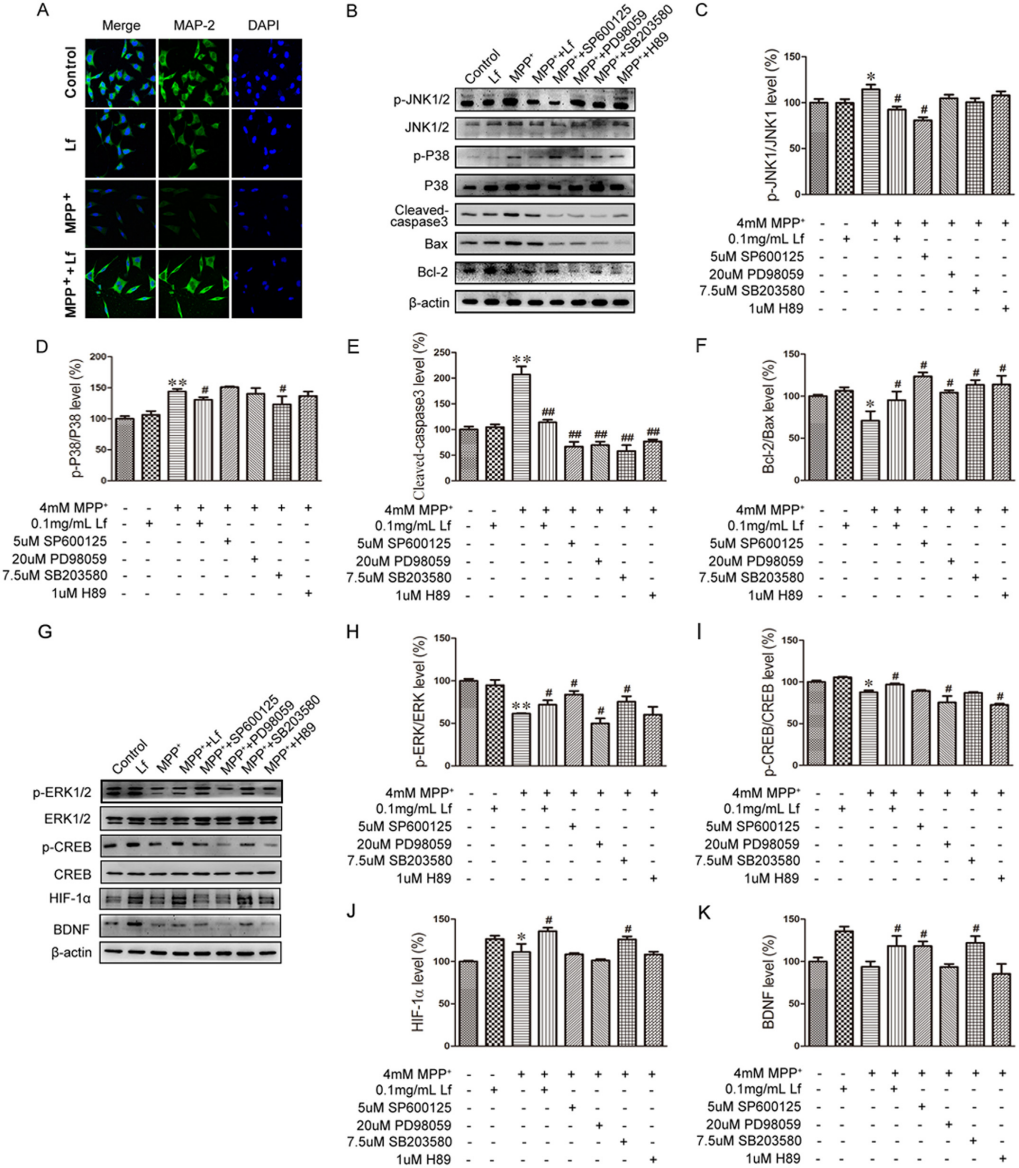
Effect of Lf on MAPK signaling pathway in SH-SY5Y cells. (A) SH-SY5Y cells were untreated or treated with 4 mM MPP^+^, 4 mM MPP^+^+0.1 mg/ml Lf, and 0.1 mg/ml Lf for 24 h. Cells were observed by fluorescence microscopy after the cells were stained with MAP-2 and DAPI. Scale bar = 50 µm. (B) The expression levels of p-JNK, JNK, p-P38, P38, cleaved-caspase3, Bax and Bcl-2 were tested by immunoblotting. Quantitative analyses of p-JNK, JNK, p-P38, P38, cleaved-caspase3, Bax and Bcl-2 are shown in C-F. (G) The expression levels of p-ERK1/2, ERK1/2, p-CREB, CREB, HIF-1α, GAP43, and BDNF were tested by immunoblotting. Quantitative analyses of p-ERK1/2, ERK1/2, p-CREB, CREB, HIF-1α, GAP43, and BDNF are shown in H-K. Values are represented as the means ± SD (n = 10). *p < 0.05, **p < 0.01 compared with the Control group; ^#^*p* < 0.05, ^##^*p* < 0.01 compared with the MPP^+^ group.

In PD model mice, we have found that hLf could attenuate the degeneration of DA neurons. To investigate the mechanisms that underlie the Lf-mediated neuroprotective effect, we treated human neuroblastoma SH-SY5Y cells with 0.1 mg/ml hLf, 4 mM MPP^+^, 4 mM MPP^+^+0.1 mg/ml hLf or homogeneous solvent for 24 h. To determine the apoptosis status, we used MAP-2 to mark the cytoplasm and synapse, and the nuclei were stained with DAPI. As shown in Fig. 6A, we found that the nucleus was normal and had a full or elliptical shape and that the cytoplasm and synapse were normal in both control and hLf-treated cells. The nucleus had a condensed state with MPP^+^-treatment compared with that of control cells, and the stability of the dendritic cytoskeleton was also changed. However, in the co-treatment cells with hLf and MPP^+^, we found that these changes were relieved.

To further clarify the Lf-mediated neuroprotective effect involved in the MAPK signaling pathway, we treated SH-SY5Y cells with SP600125, PD98059, SB203580 and H89, inhibitors of JNK1/2, ERK1/2, P38 and CREB, respectively, prior to MPP^+^ treatment. As indicated in Fig. 6B, the levels of p-JNK/JNK, p-P38/P38, and cleaved-caspase3 were significantly increased in the MPP^+^ cells compared with the controls, and the increase significantly declined in the MPP^+^+Lf cells compared with the MPP^+^ cells (p < 0.05, Fig. 6B-E). Moreover, the Bcl-2/Bax ratio showed an opposite trend compared with the expression level of cleaved-caspase3 among the groups (p < 0.05, Fig. 6B, F). Moreover, all previously described changes following hLf treatment were consistent with the effects of both the JNK inhibitor (SP600125) and P38 inhibitor (SB203580) (p < 0.05, Fig. 6B-F), which suggests that hLf attenuates MPP^+^-induced SH-SY5Y cell death by inhibiting the activation of JNK and P38.

Interestingly, the levels of p-ERK/ERK and p-CREB/CREB were markedly reduced in the MPP^+^ cells compared with the controls; however, the decrease significantly recovered after hLf treatment compared with the MPP^+^ cells (p < 0.05, Fig. 6G-I). Although there were no statistically significant differences (P > 0.05, Fig. 6G-I), the levels of p-ERK/ERK and p-CREB/CREB were reduced by pretreatment with a specific ERK or CREB inhibitor, PD98059 or H89, compared with the MPP^+^ cells. However, in the MPP^+^+SP600125 and MPP^+^+SB203580 cells, the levels of p-ERK/ERK and p-CREB/CREB were significantly increased compared with the MPP^+^ cells (p < 0.05, Fig. 6G-I). As expected, we also detected elevated HIF-1α and reduced BDNF in the MPP^+^ group relative to the controls, whereas both HIF-1α and BDNF were significantly increased compared with the MPP^+^ cells after hLf treatment (p < 0.05, Fig. 6G, J, K). In contrast, pretreatment of the cultures with PD98059 or H89 not only blocked ERK1/2 or CREB activation but also fully prevented the increase of HIF-1α and BDNF (p < 0.05, Fig. 6G, J, K), which suggests that Lf-mediated neuroprotection is involved in the upregulation of HIF-1α and BDNF via the ERK/CREB pathway in SH-SY5Y cells.

### 3.7 Lf attenuates MPP^+^-induced cell apoptosis by activating HIF-1α protein in SH-SY5Y cells and MN9D cells

**Fig. 7:**
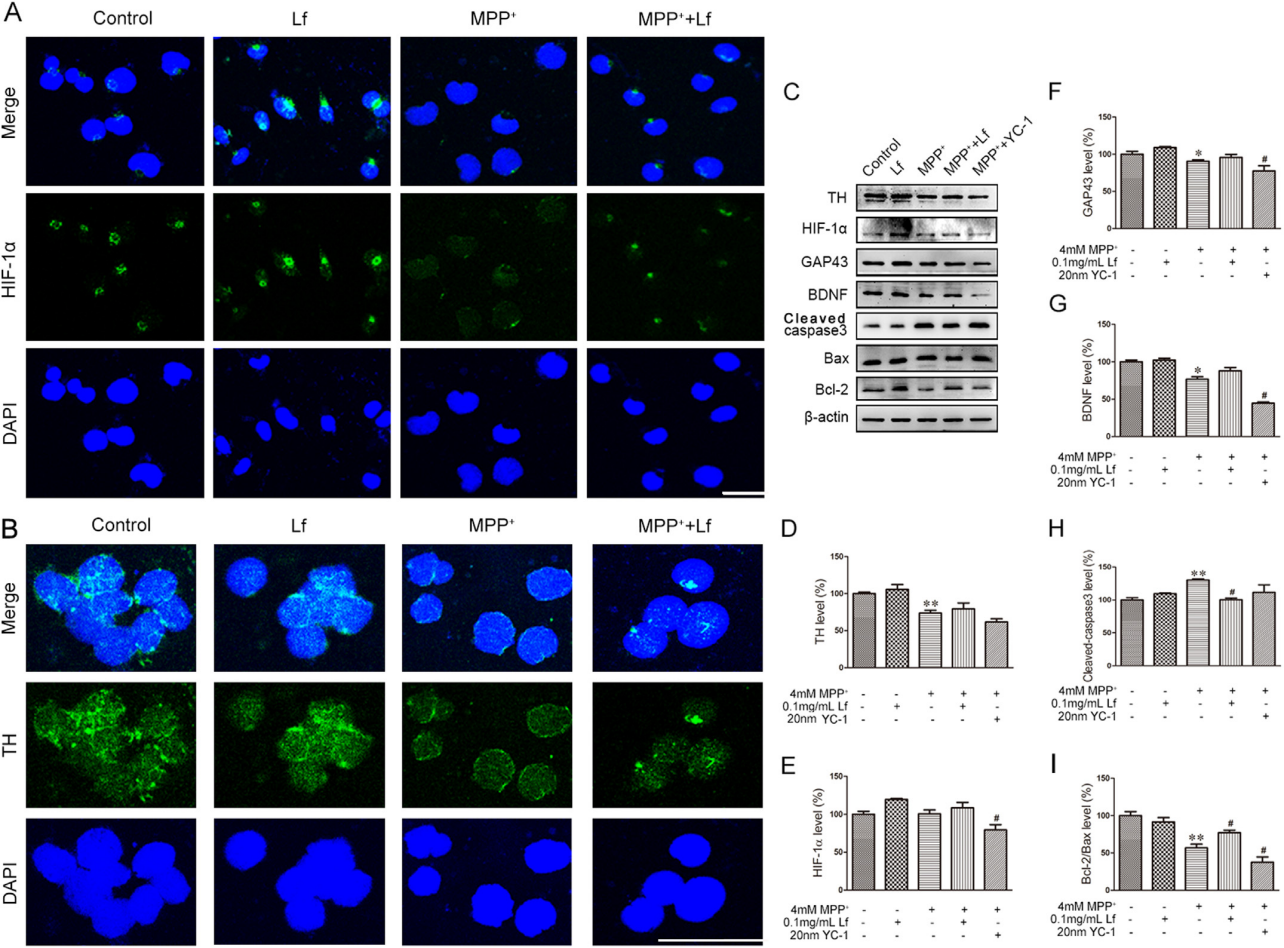
Lf attenuates MPP^+^-induced cell apoptosis by activating HIF-1α protein in SH-SY5Y cells and MN9D cells. (A, B) SH-SY5Y and MN9D cells were untreated or treated with 4 mM MPP^+^, 4 mM MPP^+^+0.1 mg/ml Lf, and 0.1 mg/ml Lf for 24 h. Cells were observed by fluorescence microscopy after the cells were stained with HIF-1α or TH following DAPI. Scale bar = 50 µm, 25 µm. (C-I) The expression levels of TH, HIF-1α, GAP43, BDNF, cleaved-caspase3, Bax and Bcl-2 in MN9D cells were tested by immunoblotting and quantitative analyses of TH, HIF-1α, GAP43, BDNF, cleaved-caspase3, Bax and Bcl-2 proteins. Values are represented as the means ± SD (n = 10). *p < 0.05, **p < 0.01 compared with the Control group; ^#^*p* < 0.05, ^##^*p* < 0.01 compared with the MPP^+^ group.

To further elucidate the protective role of HIF-1α protein in the regulation of MPP^+^-induced cell damage, we performed immunofluorescence. As shown in Fig. 7A, HIF-1α was primarily located in the nuclei of the control cells and hLf-treated cells, whereas the immunoreactivities of HIF-1α were substantially expressed in the cytoplasm of the MPP^+^-treated SH-SY5Y cells. Interestingly, in cells that underwent co-treatment with hLf and MPP^+^, the immunoreactive intensity of HIF-1α was increased compared with that of the MPP^+^ group, and a positive HIF-1α signal was found in the nuclear fraction of SH-SY5Y cells, as in control cells. Accordingly, we also observed that the expression levels of TH were significantly reduced in the MPP^+^ cells compared with the controls, whereas the TH positive fluorescence signal was stronger after hLf treatment than in the MPP^+^-treated MN9D cells (Fig. 7B).

To further determine whether the accumulated HIF-1α due to hLf attenuates MPP^+^-induced cell apoptosis, we pretreated the MPP^+^-treated MN9D cells with the HIF-1α inhibitor YC-1 in the presence or absence of hLf. As shown in Fig. 7C, the expression level of TH was significantly reduced in the MPP^+^ cells compared with the controls, and the decrease was significantly inhibited by hLf pretreatment (p < 0.01, Fig. 7C, D). In contrast, the level of TH was extremely reduced in the MPP^+^+YC-1 cells compared with the MPP^+^ cells (p < 0.05, Fig. 7C, D), which indicates hLf-induced TH upregulation via activated HIF-1α. Consistent with these results, several neuroprotective related factors, such as BDNF, GAP43, and Bcl-2, were significantly reduced accompanying the increased Bax and cleaved-caspase3, although the expression level of HIF-1α was not significantly changed in the MPP^+^-treated cells compared to the control cells. However, the levels of HIF-1α, GAP43, BDNF, and the Bcl-2/Bax ratio were significantly upregulated and the expression of cleaved-caspase3 was significantly decreased in the MPP^+^+Lf cells compared with the MPP^+^-treated cells (p < 0.05, Fig. 7C, E-I). Moreover, pretreatment of the cultures with the specific HIF-1α inhibitor YC-1 not only blocked HIF-1α but also fully prevented GAP43, BDNF, and Bcl-2. Thus, we could reasonably postulate that Lf can protect DA cells against MPP^+^-induced injury by activating the HIF-1α protein in MN9D cells.

## 4 Discussion

Our previous studies have shown that DFO, a classic iron chelator, ameliorated the pathological changes and improved behavioral disorders in animal models of AD and PD [4], [17]. In agreement with these findings, our results showed that hLf could effectively improve the movement and behavior disorders caused by MPTP, which supports the potential use of Lf as a therapeutic for PD. We subsequently assessed the in vivo neuroprotective potential of Lf in a MPTP-induced mouse model, in which the PD mouse model can replicate PD-like symptoms. Interestingly, some of these behavioral results have emerged that the MPTP-induced mice performed better after treatment with Lf compared to the control mice (Fig. 1B, D). To date, it is still not clear about the underlying mechanisms for the improved performance in the Lf treated mice, recent studies showed that Lf supplementation could improve subsequent cognitive performance during stress [36], improve spatial, association learning and memory in the supplemented animals compared to controls [37]. Importantly these studies also looked at genomic changes induced by Lf including decreased anxiety gene in the central nervous system [38], [39]. Taken together, all these imply that Lf may act via multi-signaling pathways on both motor function and non-motor function, such as cognition, learning and memory.

TH and DAT are markers for the detection of DA neurons in the SN and striatum [40], [41]. As expected, we detected that hLf can effectively alleviate the MPTP-induced loss of TH-positive neurons in the nigrostriatal area of the PD mice, whereas the recovery levels of TH and DAT by hLf treatment are inconsistent with the expected efficacy. The SH-SY5Y and MN9D cell lines were employed to induce dopaminergic neuronal death and investigate the mechanisms of MPP^+^-induced neurotoxicity [42], [43]. MAP-2 and DAPI co-staining indicated that hLf inhibited the MPP^+^-induced stability of dendritic cytoskeleton destruction and nuclear condensation in cultured SH-SY5Y cells; moreover, we confirmed that Lf could alleviate the loss of DA cells in the MN9D cell PD model. Furthermore, immunoblotting assays indicated that Lf could increase the in vivo and in vitro Bcl-2/Bax ratios, as well as decrease the expression of cleaved-caspase3, which suggests a decrease in apoptosis [44]. These data suggest that the therapeutic potentials of Lf for mitigating DA neuron/cell damage induced by MPTP/MPP^+^ deserve further investigation.

As iron chelation is one of the key functionalities of Lf [45], we determined whether Lf alleviates the MPTP-induced DA neuron damage involved in improving the iron homeostasis. Iron staining showed that Lf reduced the deposition of iron in neurons in the SN region of PD mice. Moreover, treatment with hLf induced a reduction in the DMT1 and TFR expression in the nigrostriatal area of PD mice as described in previous reports [13], which suggests that the protective effects of Lf may be due, in part, to iron chelation with a decrease of iron intake. However, it should be noted that the expression levels of Fpn do not change after hLf treatment in the brains of PD mice. Importantly, it has been reported that there were no differences between apo-Lf and holo-Lf on the neuroprotective function against MPP^+^ toxicity [46]. Thus, the protective action of Lf could not be completely explained by an effect on iron chelation in this paradigm. Moreover, except alterations in iron metabolism, the nigrostriatal toxicity of MPTP is mediated, in part, via excessive oxidative stress, an augmentation of the inflammation process, and cytotoxicity was decreased in the presence of antioxidants [47]. Interestingly, previous studies have indicated that Lf aggregates in inflammatory areas of multiple neurodegenerative diseases and Lf levels are higher in dopamine neurons in the PD brains [48]. Moreover, one of the first identified biological functions of Lf is the anti-inflammatory effect [49], and its antioxidant activities have also been widely concerned [50], [51]. This study provides good proof that Lf can inhibit MPTP-induced oxidative stress and neuroinflammation, and it showed the expressions of the antioxidant enzymes SOD1 and GPX4 were upregulated, while the ROS content and the expressions of the gliocyte markers GFAP and Iba1 were decreased; moreover, there was higher NF-κB activity and the expressions of proinflammatory TNF-α and IL-1β were decreased in the nigrostriatal tissues of PD mice following hLf treatments. This series of data is consistent with previous reports in vitro [52], which indicates that the neuroprotective function of Lf is not only dependent on its iron chelation cooperation but is also multi-functional.

Growing evidence in favor of HIF-1α being the key factor in the mechanism of neuroprotection used by chelators, including Lf [13], [53], we subsequently assessed the expression and activation of HIF-1α protein and its signal system in the PD mouse brains in both hLf- and vehicle-treated mice. We found that VEGF and BDNF were markedly increased and α-Syn was over-expressed in PD mouse brains. This dysregulation was completely corrected with the upregulation of HIF-1α after hLf treatments. It is not surprising that Lf administration stimulated a significant increase of HIF-1α [54]; however, the levels of HIF-1α and BDNF were not decreased with intracellular excess iron deposition in the nigrostriatal tissues of mice following MPTP treatments. In fact, earlier studies have suggested that a single dose of MPTP administration inhibited complex I activity and HIF-1α protein accumulation in the striatum in response to a subsequent hypoxic challenge [55], and also caused significant increases in ROS [56]. This is thought to be the main cause of MPTP-induced toxicity on dopaminergic neurons. Because the ROS is one of the major stimuli for HIF-1α expression [57], it is possible that HIF-1α, which is increased in our subacutely MPTP-induced PD mice, was induced by MPTP. Interestingly, MPP^+^ may trigger a relatively rapid induction of heme oxygenase-1 (HO-1, a HIF-dependent genes) in PC-12 cells, which HO-1 expression might have cytoprotective effects against MPP^+^-induced cytotoxicity [58]. In addition, in consistence to our data, a study revealed that chronic MPTP treatment produced a decreased TH mRNA level, but an increased BDNF mRNA level in the SN [59]. However, we found no statistical differences in the expression and activation of ERK and CREB between the controls and MPTP-treated mice, meanwhile we also observed a trend toward decrease for phospho-ERK1/2 and phospho-CREB in MPP^+^-treated cells compared with control cells, indicating that the upregulated BDNF does not necessarily bind to its receptor in MPTP-induced mice. These results together suggest that a more abundant HIF-1α and BDNF protein level may, at least partially, explain the differential vulnerability of DA neurons to MPTP and MPP^+^ toxicity.

Note that the immunoreactivities of HIF-1α were substantially expressed in the cytoplasm of MPP^+^-treated SH-SY5Y cells, whereas the expression of HIF-1α was substantially enhanced and primarily located in the nuclei of cells after hLf treatment, which suggests an increased activity. BDNF has been reported to be crucial for the survival of DA neurons [60], [61]. Preliminary evidence has implicated the ERK-CREB and HIF-1α pathways in the regulation of Lf-induced neural protection and behavioral improvement [13]. Here, we found that PD mice and cells exposed to hLf showed increases in the levels of TH, GAP43, and MAP2 (which are neurite outgrowth and differentiation markers [62]), together with ERK and CREB activation. Although the direct mechanism by which these changes occur is unknown, our previous work [4], [13] and the present data supported that HIF-1α, which is upstream of ERK-CREB, is transported to the nucleus and that HIF-1α activation by BDNF is necessary for rescuing damaged DA neurons. Nevertheless, whether the effect of Lf is related to blocking apoptotic signaling should clearly be further investigated. Studies have shown that the activation of the JNK and P38 signaling pathways is closely related to the apoptosis of DA neurons [63], and the activation of ERK contributes to protecting neurons from oxidative stress-induced apoptosis [64]. In this study, we found that the phosphorylation levels of JNK and P38 MAPK in the PD model were significantly increased, and the increase was clearly inhibited following hLf treatment. Moreover, we also confirmed that Lf not only inhibited the high expression of cleaved caspase-3 but also elevated the Bcl-2/Bax ratios in PD mice, which have been widely used to measure the degree of apoptosis [44], thus indicating that Lf inhibited cell apoptosis by blocking the JNK and P38 MAPK signaling pathways. To further clarify whether the effect of Lf on DA neuron degeneration occurs via inhibiting the activation of the JNK and P38 MAPK signaling pathways and upregulating HIF-1α via activating the ERK/CREB signaling pathway, parallel experiments involving pretreatments with hLf and inhibitors for JNK1/2, ERK1/2, P38 and CREB were performed in our PD cell model. The observation was consistent with our data in the PD mouse model that the neuroprotective effects of Lf might due to both inhibiting the JNK/p38 and apoptosis pathways and activating the ERK-CREB-BDNF and HIF-1α pathways.

In summary, our findings indicate that Lf provides neuroprotection/neurorescue against DA neuronal impairment via several mechanisms. These mechanisms may be due to not only the regulation of iron metabolism but may also rely on the inhibition of oxidative stress, neuroinflammation, apoptosis, as well as activation of the HIF-1α signaling pathway and BDNF, which subsequently relieve the symptoms of PD mice.
